# Tolerability and Safety of a Novel Ketogenic Ester, Bis-Hexanoyl (R)-1,3-Butanediol: A Randomized Controlled Trial in Healthy Adults

**DOI:** 10.3390/nu13062066

**Published:** 2021-06-16

**Authors:** Oliver Chen, Traci M. Blonquist, Eunice Mah, Kristen Sanoshy, Dawn Beckman, Kristin M. Nieman, Barbara L. Winters, Joshua C. Anthony, Eric Verdin, John C. Newman, Brianna J. Stubbs

**Affiliations:** 1 Biofortis Research, Addison, IL 60101, USA; oliver.chen@mxns.com (O.C.); traci.blonquist@mxns.com (T.M.B.); eunice.mah@mxns.com (E.M.); kristen.sanoshy@mxns.com (K.S.); Dawn.Beckman@mxns.com (D.B.); 2 Katalyses, Ankeny, IA 50023, USA; knieman@katalyses.com; 3 Winters Nutrition Associates, S Abington Township, PA 18411, USA; blwinters13@gmail.com; 4 Nlumn LLC, Princeton, NJ 08543, USA; josh@nlumn.com or; 5 Juvenescence Ltd., Princeton, NJ 08540, USA; 6 Buck Institute for Research on Aging, Novato, CA 94945, USA; everdin@buckinstitute.org (E.V.); JNewman@buckinstitute.org (J.C.N.); 7 Division of Geriatrics, UCSF, San Francisco, CA 94143, USA

**Keywords:** ketones, ketone ester, ketone diester, exogenous ketone, beta-hydroxybutyrate, gastrointestinal symptom

## Abstract

Nutritional ketosis is a state of mildly elevated blood ketone concentrations resulting from dietary changes (e.g., fasting or reduced carbohydrate intake) or exogenous ketone consumption. In this study, we determined the tolerability and safety of a novel exogenous ketone diester, bis-hexanoyl-(R)-1,3-butanediol (BH-BD), in a 28-day, randomized, double-blind, placebo-controlled, parallel trial (NCT04707989). Healthy adults (*n* = 59, mean (SD), age: 42.8 (13.4) y, body mass index: 27.8 (3.9) kg/m^2^) were randomized to consume a beverage containing 12.5 g (Days 0–7) and 25 g (Days 7–28) of BH-BD or a taste-matched placebo daily with breakfast. Tolerability, stimulation, and sedation were assessed daily by standardized questionnaires, and blood and urine samples were collected at Days 0, 7, 14, and 28 for safety assessment. There were no differences in at-home composite systemic and gastrointestinal tolerability scores between BH-BD and placebo at any time in the study, or in acute tolerability measured 1-h post-consumption in-clinic. Weekly at-home composite tolerability scores did not change when BH-BD servings were doubled. At-home scores for stimulation and sedation did not differ between groups. BH-BD significantly increased blood ketone concentrations 1-h post-consumption. No clinically meaningful changes in safety measures including vital signs and clinical laboratory measurements were detected within or between groups. These results support the overall tolerability and safety of consumption of up to 25 g/day BH-BD.

## 1. Introduction

**Figure 1:**
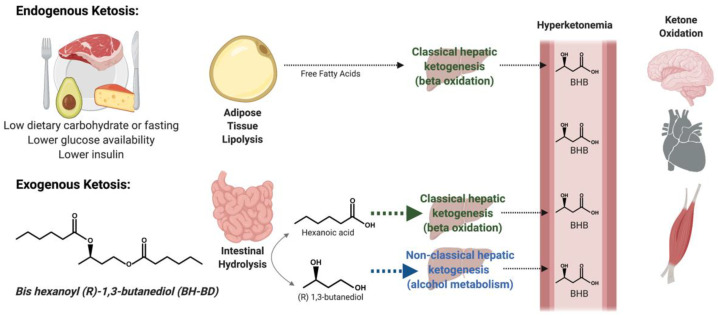
Schematic illustrating endogenous and exogenous ketosis and bis-hexanoyl-(R)-1,3-butanediol (BH-BD) metabolism. Created with Biorender.Com. Abbreviations: BHB, beta-hydroxybutyrate.

Ketosis is a metabolic state characterized by the presence of elevated blood concentrations of ketone bodies (beta-hydroxybutyrate, acetoacetate and acetone; also known as ‘ketones’) over 0.5 mM of beta-hydroxybutyrate (BHB). Ketosis occurs as a result of changes in macronutrient availability for energy metabolism (i.e., low glucose and high free fatty acids) and hormonal signaling (i.e., low insulin, high glucagon, and cortisol) [1]. The metabolic events that generate ketosis involve an increase in adipose tissue lipolysis and subsequent hepatic ketogenesis, ketone release into the circulation leading to hyperketonemia, and culminates with an increase in ketone oxidation in peripheral tissues (Figure 1).

Endogenous ketosis occurs during starvation and fasting [2], a ketogenic (low carbohydrate, high fat) diet [3] or after prolonged strenuous exercise with low carbohydrate intake [4]. In these states, glucose availability is limited and ketones provide an alternative fuel source for the brain and other peripheral tissues [5]. Furthermore, it is increasingly understood that ketones function as a signal that link the environment (i.e., carbohydrate availability) to diverse molecular responses (i.e., oxidative stress and inflammatory pathways), increasing resilience [6]. Importantly, ‘physiological’ levels of BHB (regulated ≤7 mM), the major circulating ketone, are safe and likely played a role in the survival of early man [7]; humans can produce ~150 g of ketone bodies each day during a prolonged fast [8]. Endogenous ketosis has historically been used to treat intractable seizures [3], and is under investigation for use in other clinical conditions such as type 2 diabetes [9,10], migraine [11,12,13], and glioblastoma [14,15]. Evidence suggests ketone bodies could be directly responsible for some of the beneficial molecular changes associated with dietary modifications (i.e., caloric restriction and time-restricted feeding) that can increase health span and lifespan across a variety of species [16].

Exogenous ketosis is a related metabolic state, where blood ketone concentrations are elevated as a result of consumption of a compound that contains ketone bodies or ketone body precursors, without the need for other nutritional interventions. Important distinctions between endogenous and exogenous ketosis include the origin of circulating ketone bodies (endogenous lipid stores vs. exogenous compounds) and co-existent changes in blood glucose and insulin concentrations. Decreased blood glucose and insulin concentrations occur as a result of the conditions that generate endogenous ketosis but not necessarily exogenous ketosis. Elevating blood ketone concentrations using an exogenous source is hypothesized to have multiple potential applications for human health [17,18]. Examples of exogenous ketones include medium chain triglycerides (MCTs), ketone salts and ketone esters, which have been studied for their ability to induce ketosis and their impact on a variety of non-therapeutic outcomes, including physical performance [19,20,21], cognitive function [22,23,24,25], blood glucose control and appetite regulation [26,27,28]. Ketone esters are of particular translational interest because they elevate blood ketone concentrations without an accompanying acid or mineral load [29]. The molecular structure of some ketone esters allows more complete replication of endogenous ketosis by providing substrates that elevate blood ketones via the classical process of hepatic ketogenesis, compared to compounds that deliver ketones directly into the circulation.

Ketone salts, MCTs, and at least one ketone ester have been widely studied in humans and shown to be safe and largely well tolerated [30,31,32]. Depending on the specific compound, formulation, dose and dosing context, there are some mild-moderate, transient side effects associated with exogenous ketone ingestion; these typically include nausea, dizziness and stomach cramping [33,34], not unlike the transient side-effects sometimes reported by individuals on a ketogenic diet [35]. However, associated side-effects have not been a barrier to the increasing use of exogenous ketones in research studies and in consumer products.

The novel ketone diester, bis-hexanoyl (R)-1,3-butanediol (BH-BD) is under investigation for use as a food ingredient to induce ketosis in humans. Structurally related to MCTs, BH-BD is rapidly metabolized in the presence of enzymes in the small intestine to generate ketogenic precursors: the medium chain fatty acid, hexanoic acid, and the ketogenic alcohol, (R)-1,3-butanediol [36] (Figure 1). Hexanoic acid is a substrate for classical ketogenesis, whereas (R)-1,3-butanediol is converted to ketones via a non-classical ketogenic pathway. Oral administration of BH-BD in rats and mice produced rapid and robust elevations in blood ketone concentrations [36]. However, little is known about the safety and tolerability of BH-BD in humans. Thus, the objective of this study was to evaluate the tolerability and safety of BH-BD consumed daily in a beverage by healthy adults at up to 25 g/day for 28 days, in comparison to a non-ketogenic placebo.

## 2. Materials and Methods

### 2.1. Study Design

**Figure 2:**
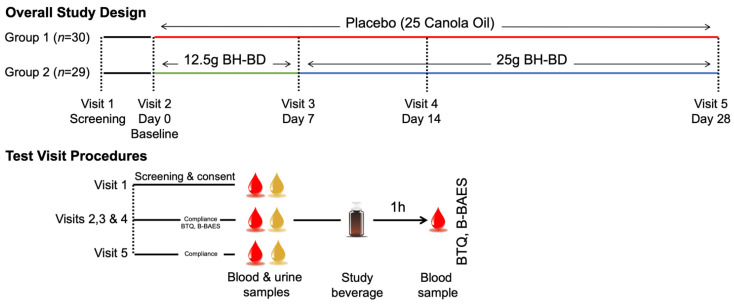
Study design schematic. Abbreviations B-BAES, Brief Biphasic Alcohol Effect Scale; BH-BD, bis-hexanoyl (R)-1,3-butanediol; BTQ, beverage tolerability questionnaire.

We conducted a randomized, double-blind, placebo-controlled, parallel study in healthy adults to evaluate the tolerability and safety of BH-BD compared to placebo (Figure 2). Subjects consumed one daily serving of BH-BD or a taste-matched placebo that contained an identical mass of non-ketogenic fat (canola oil) formulated into a 75 mL beverage, along with breakfast for 28 days. Subjects completed a Beverage Tolerability Questionnaire (BTQ) and the Brief Biphasic Alcohol Effect Scale (B-BAES) before and after consuming the study beverage each day to assess the effects of the study beverage. An institutional review board (IntegReview IRB, Austin, TX, USA, 8 December 2020, BIO-2103) approved all study related material including the protocol and informed consent documents prior to initiation of the study. Signed informed consent and authorization for use of protected health information was provided by the participants prior to implementing any protocol-specific procedures. The study was registered in the clinicaltrials.gov database as NCT04707989 and this analysis focused on BH-BD vs placebo outcomes. The study was conducted in accordance with Good Clinical Practice Guidelines, the Declaration of Helsinki [37] and United State Code of Federal Regulation Title 21. Studies took place at Biofortis Research (Addison, IL, USA) between December 2020–February 2021.

### 2.2. Participants and Screening

Participants were healthy, aged 18–65 years, BMI 18.5–34.9 kg/m^2^ and had no history of major illness, no clinically important gastrointestinal conditions, were not pregnant and were using contraception to prevent pregnancy (females only), with no recent antibiotic use (<30 days of Visit 1), no dietary habits related to ketosis (i.e., intermittent fasting, ketogenic diet), no recent use of medications known to influence gastrointestinal function, no recent use of ketone supplements, and no known allergies to any of the study beverage ingredients (including soy and milk protein). At the screening visit, participants completed a medical history questionnaire in addition to assessment of height, weight, BMI, vital signs, last menses (females only), current medication/supplement use, and review of inclusion/exclusion criteria to determine eligibility. The full inclusion and exclusion criteria can be found in the Supplemental Information. In addition, fasting blood samples were collected for analysis of clinical chemistry, hematology, lipid profile, thyroid hormones, and a urine sample was collected for urinalysis to confirm eligibility. Females under the age of 60 years completed a urine pregnancy test. Subjects had the opportunity to taste the study beverages (5–15 mL) to evaluate palatability. Subjects were then randomized to one of the study groups, based on a statistician-generated allocation sequence using a permuted-block algorithm in SAS PROC PLAN, stratified by sex. The sequence was uploaded onto the electronic data capture (EDC) platform (Medrio Inc., San Francisco, CA, USA). Subjects were asked to maintain habitual exercise, meal/diet and medication/supplementation use during the study.

### 2.3. Study Beverages

The ketone ester, BH-BD, was manufactured by Abitec Corporation (Janesville, WI, USA). All study beverages were manufactured under aseptic conditions by The National Food Laboratory (Ithaca, NY, USA). BH-BD was formulated into a chocolate flavored beverage matrix (water, whey protein concentrate, modified gum acacia, natural and artificial flavors, cocoa powder) with a total volume of 75 mL. In the first 7 days, beverages contained 12.5 g of BH-BD and 12.5 g of canola oil. For Days 8–28, study beverages contained 25 g of BH-BD. The 25 g dose was chosen to match typical single serving sizes of ketone ester and medium chain triglycerides. Placebo beverages were matched for volume and flavor using an identical chocolate flavored formulation matrix to the ketone ester beverages with the addition of a bitter additive (sucrose octaacetate) and 25 g of canola oil. Nutritional facts for study beverages are shown in Table 1. Beverages were provided as single serving bottles, labeled with the coded group allocation. All personnel involved with the data collection, analysis, and interpretation were blinded to the beverage assigned to participants. Compliance was assessed on Days 7, 14, and 28 by asking participants to return any unused product. Subjects were also instructed to complete a daily Study Beverage Log, a procedure used to increase the compliance.

### 2.4. Standard Breakfast Meal

Participants were instructed to consume the study beverage daily with breakfast. Subjects were provided a supply of breakfast foods for at-home consumption throughout the study. Breakfast meals were selected to provide a degree of standardization in conjunction with sufficient choice to maintain compliance and to replicate expected consumer use. Participants were asked to choose and consume 1–3 servings of the following foods each morning: Quaker Real Medleys Oatmeal (Summer Berry, 70 g), Jimmy Dean Delights Sandwich (Turkey Sausage, Egg Whites, & Cheese, 143 g), Kellogg’s Nutrigrain Soft Baked Breakfast Bars (Apple Cinnamon, 2 bars, 74 g) and Three Bridges Spinach and Bell Pepper Egg White Bites (2 bites, 130 g). Breakfast choice, number of servings, and any additions were recorded in the Study Beverage Log.

### 2.5. Study Questionnaires

#### 2.5.1. Study Beverage Log

The Study Beverage Log queried compliance with daily beverage and breakfast intake. Participants noted if they had consumed the beverage and breakfast, identified the number of servings of each breakfast food and any additions to the meal, the time of consumption and noted if they had experienced any adverse events. Participants completed the Study Beverage Log once daily, through the EDC platform.

#### 2.5.2. Beverage Tolerability Questionnaire (BTQ)

The BTQ used in this study was similar to that used in previous tolerability studies [38,39]. Ten tolerability issues were included in the BTQ: gas/flatulence, nausea, vomiting, abdominal cramping, stomach rumbling, burping, reflux (heartburn), diarrhea, headache, and dizziness. Firstly, participants were asked if the issue was present (pre-beverage) or had occurred since they took the study beverage (post-beverage) at the following intensities: none, mild (awareness of symptoms but easily tolerated), moderate (discomfort enough to interfere with but not prevent daily activity) or severe (unable to perform usual activity). These corresponded to scores of 0–3, respectively for each issue, giving a maximal composite score, defined as the sum of the items, of 30. Secondly, participants were asked if the frequency of the issue was: usual, somewhat more than usual or much more than usual, which corresponded to scores of 0–2, respectively. Responses were recorded through the EDC platform.

#### 2.5.3. Brief Biphasic Alcohol Effect Scale (B-BAES)

Some people report subjective positive (‘keto-buzz’) and negative (‘keto-flu’) experiences while following the ketogenic diet or after consuming exogenous ketones. Thus, we attempted to quantify these subjective sensations using B-BAES, a validated, shortened version of the Biphasic Alcohol Effect Scale (BAES) [40,41]. It consists of two subscales each comprising three items assessing either stimulation (energized, excited, up) and sedation (sedated, slow thoughts, sluggish). Participants rated how those terms described their feelings between 0 (not at all)—10 (extremely), for a maximal composite score of 30 for each subscale. Participants completed the B-BAES in the clinic pre- and 1 h post-beverage, and at home pre- and 3–6 h post beverage. Responses were recorded through the EDC platform.

### 2.6. In-Clinic Procedures

For all testing visits on Days 0, 7, and 14, subjects reported to the clinic after an overnight fast and having avoided exercise, alcohol and cannabis products for at least 10 h. On arrival, vital signs (seated, resting blood pressure, and heart rate) were measured using an automated device, body weight, last menses query (females only), and current medication/supplement use were assessed, and compliance with study instructions and inclusion/exclusion criteria was reviewed. Prior to any sampling, subjects completed a baseline BTQ and B-BAES questionnaire. Then, fasting blood samples were collected and processed for the following analysis: clinical chemistry, hematology, lipid profile (including apolipoprotein B), thyroid hormones, glucose, and BHB. A urine sample was also collected for urinalysis. After sampling, subjects were given a standard breakfast meal. Within 5 min of finishing the food, the study beverage was consumed. At 60 min after consumption of the study beverage, participants completed a second BTQ and B-BAES questionnaire and then a blood sample was collected for glucose and BHB analyses. Any adverse events (AEs) occurring during the visit were assessed. On Days 0, 7, and 14, participants were provided with a supply of study product and standard breakfast meals to take home. On Day 28, participants returned to the clinic for assessment of vitals and fasting blood and urine sample collection only, as described above.

### 2.7. At-Home Procedures

Each day at home, participants consumed a standard breakfast meal at a similar time established in-clinic and consumed the study beverage within 5 min of finishing breakfast. Participants completed the BTQ and B-BAES twice, firstly before they consumed the standard breakfast meal and study beverage and secondly immediately before they consume lunch or a snack (3–6 h after the study beverage). At breakfast, participants also completed the Study Beverage Log, which included a question to assess AEs over the last 24 h.

### 2.8. Biological Sample Analysis

All blood and urine sample analysis took place at Elmhurst Memorial Reference Laboratory (Elmhurst, IL, USA). The clinical chemistry profile included, albumin, aspartate aminotransferase, alanine aminotransferase, alkaline phosphatase, total bilirubin, calcium, chloride, creatinine, blood urea nitrogen, potassium, sodium, total protein, carbon dioxide, osmolality, glucose, and thyroid hormones. Glucose concentrations were assessed using the glucose-hexokinase glucose-6-phospahte dehydrogenase method [42] on the Dimension Vista System (Siemens Healthcare, Erlangen, Germany). Serum chemistry concentrations were assessed using the Dimension Vista system. The hematology profile included white blood cell count, red blood cell count, hemoglobin concentration, hematocrit (as volume percent), mean corpuscular volume, mean corpuscular hemoglobin concentration, neutrophils, lymphocytes, monocytes, eosinophils, basophils and platelet count. Urinalysis parameters included glucose, urobilinogen, ketone body, protein, blood cells, bilirubin, specific gravity, and pH. Urinalysis was conducted using IRIS test strip (Mumbai, India) and refractive index with specific gravity, transmitted light for color, scattered light for clarity, and digital camera for cells, crystals, and organisms. TSH and T3 were analyzed by chemiluminescent immunoassays using Dimension Vista system and ADIVA Centaur system (Siemens Medical Solutions, Malvern, PA, USA), respectively. T4 was measured using an emit homogeneous enzyme immunoassay (Dimension Vista). Levels of total cholesterol, high-density lipoprotein cholesterol (HDL-C), triglycerides, non-HDL-C were determined using the Dimension Vista system. Low-density lipoprotein cholesterol was calculated according to the Friedewald equation [43]. Apolipoprotein B was measured using quantitative nephelometry. BHB was assessed using an enzymatic colormetric assay (EKF Diagnostics—Stanbio Labs, Boerne, TX, USA) on the Cobas C510 analyzer (Roche Diagnostics, Indianapolis, IN, USA). Normal ranges for all values were provided by the analytical lab and accounted for age and gender.

### 2.9. Statistical Methods

The study was designed to have 70% power at a two-sided 0.05 significance level to detect a 2-fold difference in the continuous composite scores of the BTQ and B-BAES subscales. Based on previous ketone ester trials [33], an evaluable sample size of ~25/group was targeted and 5 additional subjects/group were enrolled to account for an estimated 20% attrition rate.

The statistical analysis plan was finalised prior to database lock and all analyses were conducted blinded. The primary outcome was the difference between groups in the daily 3–6 h post-consumption composite score of issue intensity over 28 days of at-home assessment between BH-BD and placebo. Due to the large number of 0 intensity events, the daily proportion of participants with at least one mild post-consumption event (composite score >0) was modelled over 28-days using a generalized linear mixed model following a binary distribution with a logit link. The initial model contained fixed effect terms for day (continuous), beverage, and the day by beverage interaction; a random intercept and slope were included. Additionally, the weekly total BTQ composite intensity score was calculated as the sum of the individual days. Missing data was imputed as the weekly average multiplied by 7. The pre- and post-consumption weekly total composite scores were compared between and within groups with the Wilcoxon rank sum and Wilcoxon signed rank test, respectively. The proportion of participants with any issue (composite score >0), was compared between groups with the Fisher’s exact test. An exploratory analysis of the individual items was conducted for which the Fisher’s exact test was used to compare the proportion of participants with (1) issue reported at least once, (2) issue reported at least twice, and (3) moderate-to-severe issue reported at least once.

The daily at-home post-consumption B-BAES subscale scores for stimulation and sedation were modelled over 28-days with a linear mixed model. The initial model contained terms for day (continuous), beverage, day by beverage interaction, and daily pre-consumption score; a random intercept and slope were included. The in-clinic B-BAES scores were similarly modelled with a repeated measures mixed model except the initial model including the clinic visit (categorical) and a random intercept. Pairwise comparisons between the beverages were estimated at each pre-specified day/clinic visit. At each clinic visit, an analysis of covariance (ANCOVA) approach was used to evaluate the beverage group differences in glucose and BHB levels 1-h post-consumption adjusting for the pre-consumption measure. The within group change in vitals and safety laboratory measures was tested with the Wilcoxon signed rank test.

Significant *p*-values (defined *a priori*) were considered at α = 0.05, two-sided. All analyses were conducted using SAS for Windows (version 9.4, Cary, NC, USA) and/or R (version 3.6.0, The R Foundation for Statistical Computing, Vienna, Austria). Data are presented as means ± standard deviation (SD) unless otherwise noted.

## 3. Results

### 3.1. Compliance and Adverse Events

Participants (*n* = 59) were randomized to the BH-BD or placebo condition, and all completed the study protocol in its entirety. Participant anthropometric characteristics at baseline are shown in Table 2. The analysis population (*n* = 59) consisted of all randomized participants and compliance with consumption of the study products for the overall population was 99.9 ± 2.2%. One study-product related AE (constipation) occurred in the placebo group; no study-product related AEs occurred in the BH-BD group.

### 3.2. Tolerability

**Figure 3:**
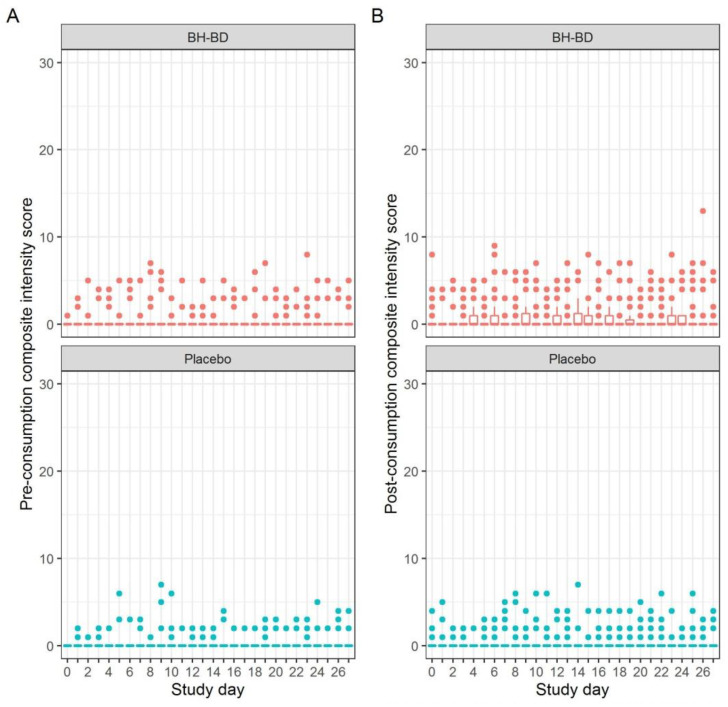
Daily beverage tolerability composite scores for healthy adults consuming up to 25 g/day of BH-BD or placebo. 10 tolerability issues (gas/flatulence, nausea, vomiting, abdominal cramping, stomach rumbling, burping, reflux/heartburn, diarrhea, headache, and dizziness) were scored 0 = none, 1 = mild, 2 = moderate and 3 = severe, giving a maximal composite score of 30. (**A**) Pre-beverage composite score; (**B**) Post-beverage composite score. Dots indicate statistical outliers identified by the 1.5 x interquartile (IQR) rule.

Analysis of the primary endpoint of long-term tolerability by at-home composite BTQ scores demonstrated no tolerability issues with up to 25 g/day of BH-BD. There was a high rate of ‘no symptoms’ reported through the study in both study groups when examined by severity (Figure 3) and when adjusted for frequency (data not shown). There were no significant differences in the daily proportion of participants with any tolerability issues over 28 days between BH-BD vs. placebo (interaction effect, *p* = 0.66). There were no significant differences within groups in the weekly composite BTQ scores, importantly highlighting no difference in tolerability between the lower (12.5 g; Days 0–6) and higher (25 g; Days 7–28) doses of BH-BD (Table 3). There was no significant difference in proportion of participants reporting any acute (in-clinic, 1-h post-beverage) tolerability issues between study groups at Days 0, 7 or 14 (Table 4).

During exploratory analysis, each tolerability issue listed on the BTQ was analyzed individually for the likelihood of occurrence on at least one day during the study at any intensity. There were no differences between BH-BD and placebo in occurrence of burping, cramping, diarrhea, gas, reflux, vomiting or rumbling. There was a significantly increased frequency of participants consuming BH-BD who reported dizziness (BH-BD = 7, placebo = 0; *p* = 0.011), nausea (BH-BD = 14, placebo = 4; *p* = 0.010), and headache (BH-BD = 14, placebo = 4; *p* = 0.010) (Table 5). The median number (Q1, Q3) of days where dizziness, headache, and nausea were reported was 0 (0, 0), 0 (0, 1), and 0 (0, 3), respectively. The third quartile (Q3) value indicates that approximately 75% of subjects had a value less than that of Q3. When this analysis was refined to examine the likelihood of tolerability issue occurrence at any intensity on two or more days during the study, both headache (BH-BD = 7, placebo = 0; *p* = 0.011), and nausea (BH-BD = 9, placebo = 2; *p* = 0.042) were more likely in the BH-BD group (Table 5). The only tolerability issue that was significantly more common with moderate-to-severe intensity on at least one day during the study in the BH-BD vs. placebo group was nausea (BH-BD = 6, placebo = 0; *p* = 0.024) (Table 5). Six subjects reported 14 moderate-severe nausea events, but only one was severe reported by one subject on day 0 (first day of product consumption).

### 3.3. Stimulation and Sedation

There was no significant difference in at-home B-BAES stimulation or sedation scores between BH-BD vs. placebo over the 28-day intervention (interaction effect = 0.11). For the acute, in-clinic B-BAES scores that were collected 1-h post-beverage consumption, there was a significantly higher sedation score and lower stimulation score on Day 0 when 12.5 g of BH-BD was administered vs. placebo that was retained when also adjusting for age and sex (1-h post-stimulation: BH-BD vs. placebo = −2.77, *p* = 0.011; 1-h post-sedation: BH-BD vs. placebo = +2.22, *p* = 0.021). Notably, this acute effect represented a small change in the 30-point composite B-BAES subscales (~1 point change from baseline). Further, significant effects were not seen during any of the subsequent clinic visits (Days 7 and 14) when 25 g of BH-BD was consumed (Table 4) or in the at-home, 3–6-h post-beverage stimulation or sedation scores.

### 3.4. Safety Laboratory Tests

Consumption of 25 g/day of BH-BD was not associated with any clinically relevant changes in safety laboratory values. There were no changes in vital signs (data not shown), body weight was unchanged in both groups (data not shown), and there were no safety concerns in any of the laboratory tests conducted: clinical chemistry, hematology, lipid panel, thyroid panel or urinalysis (Supplementary Tables S1–S5). Initial analyses focused on within-group changes in laboratory values between Days 0, 7, 14, and 28 found no consistent changes over the course of the study; subsequent analysis of differences between BH-BD vs. placebo found no parameters that were consistently different during the study (data not shown).

### 3.5. Acute Glucose and Ketone Measurement

There was a highly significant increase in circulating BHB concentrations 1-h post-BH-BD beverage consumption compared to placebo on Days 0, 7 and 14 (Table 6). Post-BH-BD, BHB concentrations were lower on Day 0, when the study beverages contained the lower dose of active ingredient (12.5 g) and were similar between Days 7 and 14 when the 25-g dose was consumed. There were no significant differences in pre- or 1-h post beverage circulating glucose concentrations between BH-BD vs. placebo on any of the clinic visits (Table 6).

## 4. Discussion

The results of this study demonstrate that daily consumption of up to 25 g of BH-BD by healthy adults for 28 consecutive days was safe and well-tolerated. Participant compliance was high, with no drop-outs and no study product-related adverse events. There was a high rate of ‘no symptoms’ of any type reported throughout the study, and notably few gastrointestinal issues. There was an increased proportion of participants who reported headache, nausea, and dizziness at least once in the BH-BD group compared to the placebo group, but the absolute incidence was low, and severity was generally mild. There were no clinically meaningful changes in laboratory results from baseline, supporting the safety of BH-BD. Finally, this study demonstrated that BH-BD successfully generated nutritional ketosis.

Whilst there were no differences in our primary outcome of BTQ composite score, it is unsurprising that BH-BD caused some mild, transient side effects in a proportion of participants. Similar side effects have been reported with consumption of other exogenous ketones or ketogenic precursors, at a range of serving sizes (10–85 g) and under different contexts (fed, fasted, rest, exercise). Side-effects of exogenous ketones may include acute gastrointestinal discomfort and distress, as well as systemic symptoms such as nausea and dizziness that range in intensity from mild to severe (Table 7). Some effects could be linked to the known bitter taste of exogenous ketones, which are difficult to mask and may lead to acute feelings of nausea. MCTs, which are structurally similar to BH-BD, can cause cramps, pain and diarrhea under some dosing contexts [34,44], therefore physicians using MCTs for disease treatment, recommend increasing serving size gradually to build tolerance [45,46], similar to the escalating serving size strategy used in this study. Overall, BH-BD was well tolerated and the limited side-effects of BH-BD intake are not interpreted to be a tolerability or safety concern.

Whilst the finding of acutely increased sedation and decreased stimulation on Day 0, when 12.5 g of BH-BD was consumed, was statistically significant, it is not interpreted to be clinically meaningful. The absolute change in B-BAES score from baseline values seen in this study following BH-BD consumption was very small (1 point of the 30-point scale) and was far smaller than seen when BAES or B-BAES is administered during alcohol induced intoxication (>5 points of the 30-point scale) [41,60,61]. The alterations seen on Day 0 were surprising given that no further effects were found either in the subsequent acute in-clinic visits or during at-home B-BAES scores when 25 g of BH-BD was consumed. It is unclear what led to the B-BAES differences observed on Day 0. Whilst unlikely given the known rapid hydrolysis of BH-BD in the intestines and plasma [36], it cannot be excluded that BH-BD or its hydrolysis or metabolic products may have had a biochemical effect and caused sedation and lower stimulation on the first occasion of consumption, which disappeared subsequently with rapid adaptation. Alternatively, other elements of the study which were new to the participants may have led to an anomalous result on Day 0, such as the first day consuming a non-habitual standard breakfast or the first time completing the B-BAES questionnaire. As B-BAES is designed to differentiate between the ascending and descending limbs of the blood alcohol curve, any possible effects of BH-BD on stimulation or sedation could be explored by collecting multiple acute B-BAES scores in future studies.

In this study, 25 g of BH-BD taken with a meal effectively raised ketone levels to generate a state of nutritional ketosis (>0.5 mM BHB) at 1-h post-consumption, but did not affect blood glucose concentrations. Limited conclusions can be drawn from this single time point regarding the dynamic effect of BH-BD on blood ketone kinetics, and work to characterize the acute blood ketone responses to BH-BD consumption is underway. As with BHB, without a full kinetic time course of blood glucose concentrations, it is unclear if BH-BD had an effect that occurred before or after the 1-h time point as the maximum postprandial blood glucose can occur as early as 30 min after a meal. Additionally, in this study the carbohydrate content of the provided breakfast choices was highly variable which might confound the observation. As ketone esters have been demonstrated to lower blood glucose responses following an oral glucose tolerance test and a mixed meal in acute and multi-day studies [26,27,28,62], the effect of BH-BD on blood glucose concentrations is a promising area for future investigation.

The strengths of this study included the duration, the sample size, the free-living design being highly relevant for future consumption of BH-BD, high compliance achieved, and the similarity between the BH-BD and placebo drinks used. Additionally, multiple approaches (acute in-clinic and chronic at-home measures) were used to confirm tolerance of BH-BD. Weaknesses included the lack of time course measurements for BHB, glucose and the B-BAES. Furthermore, as participants were required to complete the BTQ daily, it is not clear if any of the side effects would have been reported spontaneously without prompting. Finally, the safety and tolerability of higher serving sizes of BH-BD remains unknown.

In conclusion, the results of this study demonstrate the safety and tolerability of up to 25 g/day of the ketone diester, BH-BD in healthy adults. These findings will be foundational for the design of future clinical research studies using BH-BD as a tool to induce a metabolic switch to nutritional ketosis and facilitate the study of ketosis for health and resilience.

## Figures and Tables

**Table 1:** Nutritional information of Study Beverages.

	Day 0–7 BH-BD	Day 8–28 BH-BD	Placebo
BH-BD (g) *	12.5	25	-
Canola oil (g)	12.5	-	25
Energy (kcal)	225	210	246
Total carbohydrate (g)	1	2	2
Total fat (g)	13	0.5	25.5
Protein (g)	1	2	2

Abbreviations: BH-BD, bis-hexanoyl (R)-1,3-butanediol.* Caloric content of BH-BD was determined to be ~7.8 kCal/g using a bomb calorimeter.

**Table 2:** Baseline demographic characteristics of randomized participants in the BH-BD and placebo condition.

	Beverage Group	
	BH-BD (*n* = 30)	Placebo (*n* = 29)
Age (years)	43.9 (11.7)	41.7 (15.0)
Sex	*n* (%)	
Female	16 (53.3)	16 (55.2)
Male	14 (46.7)	13 (44.8)
Race	*n* (%)	
Asian	4 (13.3%)	2 (6.9%)
Black/African American	3 (10.0%)	3 (10.3%)
Native Hawaiian or Other Pacific Islander	1 (3.3%)	0 (0%)
White	22 (73.3%)	23 (79.3%)
Multiracial	0 (0%)	1 (3.4%)
Anthropometrics	Mean (SD)	
Height (cm)	168.5 (10.6)	171.0 (11.6)
BMI (kg/m^2^)	27.9 (3.8)	27.7 (4.0)
Weight (kg)	79.9 (17.8)	8281.5 (16.9)

Abbreviations: BH-BD, bis-hexanoyl (R)-1,3-butanediol; BMI, body mass index; SD, standard deviation.

**Table 3:** Weekly total composite BTQ scores for healthy participants either before or 3–6 h after consuming BH-BD or placebo at home.

Days (BH-BD per Serving)	Timing	Beverage Group	Weekly Total Composite BTQ Score ^1^		Change vs. Previous Week	
			Mean (SD)	*p*-Value ^2^	Mean (SD)	*p*-Value ^3^
0–6 (12.5 g)	Before	BH-BD	2.4 (6.2)	0.47	-	-
		PLA	0.9 (1.9)		-	-
	After	BH-BD	4.7 (7.9)	0.16	-	-
		PLA	2.0 (4.2)		-	-
7–13 (25 g)	Before	BH-BD	2.7 (6.1)	0.42	0.3 (4.6)	0.29
		PLA	1.5 (2.6)		0.6 (2.3)	0.27
	After	BH-BD	5.6 (9.3)	0.29	0.9 (6.9)	0.57
		PLA	3.4 (6.9)		1.3 (6.1)	0.49
14–22 (25 g)	Before	BH-BD	2.3 (5.4)	0.40	−0.4 (2.6)	0.26
		PLA	1.2 (3.3)		−0.3 (2.2)	0.52
	After	BH-BD	5.5 (10.0)	0.32	−0.1 (3.4)	0.72
		PLA	2.4 (6.5)		−0.9 (3.4)	0.18
21–27 (25 g)	Before	BH-BD	2.2 (5.3)	0.44	−0.1 (2.4)	0.85
		PLA	2.0 (4.7)		0.8 (2.7)	0.13
	After	BH-BD	6.2 (12.3)	0.30	0.6 (5.0)	0.43
		PLA	3.0 (6.7)		0.6 (3.4)	0.65

Abbreviations: BH-BD, bis-hexanoyl (R)-1,3-butanediol, BTQ, beverage tolerability questionnaire; PLA, placebo; SD, standard deviation. ^1^ The weekly total BTQ composite intensity score was calculated as the sum of the individual days (week 1: day 0–6, week 2: day 7–13, week 3: day 14–20, week 4: day 21–27). ^2^ The Wilcoxon rank sum test was utilized, and *p*-values presented for BH-BD vs. placebo. ^3^ The Wilcoxon signed rank test was utilized, and *p*-values presented for within group comparisons.

**Table 4:** BTQ composite score and B-BAES stimulation and sedation scores, prior to and 1-h post study beverage consumption during clinic visits on Days 0, 7, and 14.

Clinic Visit Day	Timing	Beverage Group	BTQ		B-BAES			
			BTQ Composite *n* (%)	*p*-Value ^1^	SED Score Mean (SD)	*p*-Value ^2^	STIM Score Mean (SD)	*p*-Value ^2^
0 ^3^	Pre-	BH-BD	4 (13.3)	0.11	4.17 (5.11)	-	19.73 (4.77)	-
		PLA	0 (0.0)		4.55 (5.09)		17.38 (6.73)	
	1-h post	BH-BD	2 (6.7)	1.00	5.57 (6.62)	-	17.13 (7.04)	-
		PLA	3 (10.3)		3.59 (4.51)		18.17 (6.80)	
	△pre vs. 1-h post	BH-BD	1 (3.3)	0.35	-	-	-	-
		PLA	3 (10.3)					
	Model Estimate △ BH-BD vs. PLA				2.22	0.021	−2.77	0.011
7 ^3^	Pre-	BH-BD	3 (10)	1.00	3.77 (5.20)	-	17.07 (7.67)	-
		PLA	2 (6.9)		3.10 (4.30)		16.38 (7.36)	
	1-h post	BH-BD	8 (26.7)	0.33	3.73 (5.34)	-	17.33 (7.49)	-
		PLA	4 (13.8)		2.03 (3.46)		18.83 (6.16)	
	△pre vs. 1-h post	BH-BD	5 (16.7)	0.71	-	-	-	-
		PLA	3 (10.3)					
	Model Estimate △ BH-BD vs. PLA				1.35	0.16	−1.91	0.075
14 ^3^	Pre-	BH-BD	2 (6.7)	0.67	3.73 (5.23)	-	17.30 (7.73)	-
		PLA	3 (10.3)		3.07 (3.99)		16.38 (6.83)	
	1-h post	BH-BD	8 (26.7)	0.33	3.70 (5.02)	-	17.70 (7.84)	-
		PLA	4 (13.8)		2.21 (3.77)		17.90 (7.04)	
	△pre vs. 1-h post	BH-BD	6 (20.0)	0.25	-	-	-	-
		PLA	2 (6.9)					
	Model Estimate △ BH-BD vs. PLA				1.15	0.23	−0.80	0.46

Abbreviations: -, not done; △, change; BH-BD, bis-hexanoyl (R)-1,3-butanediol; B-BAES, brief biphasic alcohol effect scale; BTQ, beverage tolerability questionnaire; SED, sedation; STIM, stimulation; SD, standard deviation; PLA, placebo; pre-, pre-consumption. On Days 7 and 14, 25 g of BH-BD was consumed. ^1^ Fisher’s exact test was utilized and *p*-values presented for BH-BD vs. placebo. ^2^ A repeated measures linear mixed model was utilized and *p*-values presented for BH-BD vs. placebo. ^3^ On Day 0, 12.5 g of BH-BD was consumed.

**Table 5:** Proportion of participants reporting each BTQ symptom on at least one or two or more days and number of participants who reported moderate or severe issues during the 28-day study.

Issue	Beverage Group	#Days (*n*) Issue Reported Mean (SD)	Proportion of Subjects with BTQ Issue				Proportion of Subjects with Moderate—Severe Issue	
			At Least Once		At Least Twice			
			*n* (%)	Overall *p*-Value ^1^	*n* (%)	Overall *p*-Value ^1^	*n* (%)	Overall *p*-Value ^1^
Burping	BH-BD	1.4 (4.6)	7 (23.3)	1.00	4 (13.3)	0.73	0 (0.0)	0.24
	PLA	2.8 (7.2)	6 (20.7)		5 (17.2)		2 (6.9)	
Cramping	BH-BD	1.2 (3.0)	10 (33.3)	0.78	4 (13.3)	1.00	4 (13.3)	0.11
	PLA	0.5 (1.0)	8 (27.6)		3 (10.3)		0 (0.0)	
Diarrhea	BH-BD	1.4 (4.0)	7 (23.3)	0.57	4 (13.3)	0.73	3 (10.0)	0.47
	PLA	0.9 (2.5)	9 (31.0)		5 (17.2)		5 (17.2)	
Dizziness	BH-BD	1.1 (3.0)	7 (23.3)	0.011	5 (16.7)	0.052	2 (6.7)	0.49
	PLA	0.0 (0.0)	0 (0.0)		0 (0.0)		0 (0.0)	
Gas	BH-BD	2.7 (5.0)	16 (53.3)	1.00	12 (40.0)	0.79	3 (10.0)	0.47
	PLA	2.4 (4.4)	16 (55.2)		13 (44.8)		5 (17.2)	
Headache	BH-BD	1.9 (4.3)	14 (46.7)	0.010	7 (23.3)	0.011	3 (10.0)	0.24
	PLA	0.1 (0.4)	4 (13.8)		0 (0.0)		0 (0.0)	
Nausea	BH-BD	2.3 (4.3)	14 (46.7)	0.010	9 (30.0)	0.042	6 (20.0)	0.024
	PLA	0.2 (0.6)	4 (13.8)		2 (6.9)		0 (0.0)	
Reflux	BH-BD	1.8 (4.7)	6 (20.0)	0.47	5 (16.7)	0.42	5 (16.7)	0.19
	PLA	0.6 (2.8)	3 (10.3)		2 (6.9)		1 (3.4)	
Rumbling	BH-BD	2.9 (5.0)	13 (43.3)	0.79	11 (36.7)	0.40	5 (16.7)	0.19
	PLA	1.6 (3.7)	11 (37.9)		7 (24.1)		1 (3.4)	
Vomiting	BH-BD	0.3 (1.6)	2 (6.7)	0.49	1 (3.3)	1.00	1 (3.3)	1.00
	PLA	0.0 (0.0)	0 (0.0)		0 (0.0)		0 (0.0)	

Abbreviations: BH-BD, bis-hexanoyl (R)-1,3-butanediol, BTQ, Beverage Tolerability Questionnaire; SD, standard deviation. ^1^ Fisher’s exact test was utilized.

**Table 6:** Serum BHB and glucose concentrations in healthy participants either before or 1 h after consuming BH-BD (*n* = 30) or placebo (*n* = 29) during clinic visits.

Clinic Visit Day	Timing	Beverage	BHB (mM)		Glucose (mg/dL)	
			Mean (SD)	*p*-Value ^2^	Mean (SD)	*p*-Value ^2^
0 ^1^	Pre-	BH-BD	0.102 (0.052)		93.40 (7.84)	
		PLA	0.128 (0.151)		91.59 (10.47)	
	1-h post	BH-BD	0.431 (0.240)	<0.001	96.97 (18.83)	0.17
		PLA	0.083 (0.050)		100.4 (24.85)	
	△pre vs. 1-h post	BH-BD	0.329 (0.219)		3.57 (14.46)	
		PLA	−0.046 (0.140)		8.79 (21.03)	
7 ^1^	Pre-	BH-BD	0.108 (0.077)		90.97 (9.02)	
		PLA	0.131 (0.106)		88.52 (7.47)	
	1-h post	BH-BD	0.985 (0.444)	<0.001	96.07 (28.24)	0.38
		PLA	0.082 (0.030)		97.00 (21.03)	
	△pre vs. 1-h post	BH-BD	0.876 (0.427)		5.10 (25.31)	
		PLA	−0.049 (0.088)		8.48 (17.16)	
14 ^1^	Pre-	BH-BD	0.104 (0.046)		92.37 (11.62)	
		PLA	0.134 (0.132)		87.07 (8.15)	
	1-h post	BH-BD	0.980 (0.392)	<0.001	93.80 (29.53)	0.092
		PLA	0.086 (0.032)		93.66 (22.98)	
	△pre vs. 1-h post	BH-BD	0.875 (0.365)		1.43 (22.84)	
		PLA	−0.048 (0.119)		6.59 (18.12)	

Abbreviations: BH-BD, bis-hexanoyl (R)-1,3-butanediol, PLA, placebo; pre-, pre-consumption; SD, standard deviation. ^1^ On Day 0, 12.5 g of BH-BD was consumed. On Days 7 and 14, 25 g of BH-BD was consumed. ^2^ An analysis of covariance model was utilized and adjusted *p*-values presented for BH-BD vs. placebo.

**Table 7:** Overview of reported gastrointestinal symptoms in studies of different exogenous ketone compounds.

Exogenous Ketone Type	Reference(s)	Participant Type (*n*)	Daily Serving Size Range	Symptoms Reported
MCT	[34,44,47,48,49]	Healthy adults before/after exercise (25) or adults with Alzheimer’s disease (260)	20–85 g	GI distress, nausea, vomiting, abdominal pain, bloating, diarrhea, flatulence, dyspepsia, dizziness, headache
Ketone Salts	[22,33,50,51,52,53,54]	Healthy adults at rest (23) or before/after exercise (44)	12–36.5 g	GI distress, nausea, diarrhea, vomiting, light-headedness, cramps
AcAc Ketone Diester	[55]	Healthy adults before exercise (11)	~37 g	Vomiting, dizziness, nausea, reflux
1,3 Butanediol	[56]	Healthy adults before exercise (9)	~24 g	Nausea, euphoria, dizziness, belching, abdominal pain
BHB Ketone Monoester	[24,33,50,57,58,59]	Healthy adults at rest (43) or before/after exercise (27)	12–155 g	Vomiting, abdominal pain, nausea, flatulence, heartburn, headache, dizziness
BH-BD	Current study	Healthy adults	12.5–25 g	Headache, nausea, dizziness

Abbreviations: MCT, medium chain triglyceride; GI, gastrointestinal, AcAc, acetoacetate, BHB, beta-hydroxybutyrate.

## Data Availability

The data presented here are available upon request from the corresponding author due to intellectual property limitations.

## Supplementary Materials

The following are available online at https://www.mdpi.com/article/10.3390/nu13062066/s1, Full lists of inclusion/exclusion and exclusionary medications/supplements/products criteria utilized, Table S1, Clinical chemistry measures at clinic visits during 28-day study of healthy adults consuming up to 25 g/day of BH-BD or placebo, Table S2, Haematology measures at clinic visits during 28-day study of healthy adults consuming up to 25 g/day of BH-BD or placebo, Table S3, Blood lipid measures at clinic visits during 28-day study of healthy adults consuming up to 25 g/day of BH-BD or placebo, Table S4, Thyroid hormone measures at clinic visits during 28-day study of healthy adults consuming up to 25 g/day of BH-BD or placebo, Table S5, Urinalysis measures at clinic visits during 28-day study of healthy adults consuming up to 25 g/day of BH-BD or placebo, Figure S1, CONSORT diagram.

## References

[1] Robinson A.M., Williamson D.H. (1980). Physiological roles of ketone bodies as substrates and signals in mammalian tissues. Physiol. Rev..

[2] Sherwood L.M., Parris E.E., Cahill G.F. (1970). Starvation in Man. N. Engl. J. Med..

[3] Gilbert D.L., Pyzik P.L., Freeman J.M. (2000). The ketogenic diet: Seizure control correlates better with serum beta-hydroxybutyrate than with urine ketones. J. Child Neurol..

[4] Koeslag J.H., Noakes T.D., Sloan A.W. (1980). Post-Exercise Ketosis. J. Physiol. Lond..

[5] Owen O.E., Morgan A.P., Kemp H.G., Sullivan J.M., Herrera M.G., Cahill G.F. (1967). Brain Metabolism during Fasting*. J. Clin. Investig..

[6] Newman J.C., Verdin E. (2014). Ketone bodies as signaling metabolites. Trends Endocrinol. Metab..

[7] Krebs H. (1966). The regulation of the release of ketone bodies by the liver. Adv. Enzym. Regul..

[8] Reichard G., Owen O.E., Haff A.C., Bortz W.M. (1974). Ketone-body production and oxidation in fasting obese humans. J. Clin. Investig..

[9] Hallberg S.J., McKenzie A.L., Williams P.T., Bhanpuri N.H., Peters A.L., Campbell W.W., Hazbun T.L., Volk B.M., McCarter J., Phinney S.D. (2018). Effectiveness and Safety of a Novel Care Model for the Management of Type 2 Diabetes at 1 Year: An Open-Label, Non-Randomized, Controlled Study. Diabetes Ther..

[10] Athinarayanan S.J., Adams R.N., Hallberg S.J., McKenzie A.L., Bhanpuri N.H., Campbell W.W., Volek J.S., Phinney S.D., McCarter J.P. (2019). Long-Term Effects of a Novel Continuous Remote Care Intervention Including Nutritional Ketosis for the Management of Type 2 Diabetes: A 2-Year Non-randomized Clinical Trial. Front. Endocrinol..

[11] Strahlman R.S. (2006). Can Ketosis Help Migraine Sufferers? A Case Report. Headache. J. Head Face Pain.

[12] Di Lorenzo C., Currà A., Sirianni G., Coppola G., Bracaglia M., Cardillo A., De Nardis L., Pierelli F. (2014). Diet transiently improves migraine in two twin sisters: Possible role of ketogenesis?. Diet Exerc. Cogn. Funct. Neurol. Dis..

[13] Di Lorenzo C., Coppola G., Sirianni G., Di Lorenzo G., Bracaglia G., Di Lenola D., Siracusano A., Rossi P., Pirelli F. (2015). Migraine improvement during short lasting ketogenesis: A proof-of-concept study. Eur. J. Neurol..

[14] Zuccoli G., Marcello N., Pisanello A., Servadei F., Vaccaro S., Mukherjee P., Seyfried T.N. (2010). Metabolic management of glioblastoma multiforme using standard therapy together with a restricted ketogenic diet: Case Report. Nutr. Metab..

[15] Van der Louw E., Olierman J.F., van den Dernt P.M.L.A., Bromberg J.E.C., de Hoop E.O., Neuteboorn R.F., Catsman-Berrevoets C.E., Vincent A.J.P.E. (2019). Ketogenic diet treatment as adjuvant to standard treatment of glioblastoma multiforme: A feasibility and safety study. Adv. Med. Oncol..

[16] Veech R.L., Bradshaw P.C., Clarke K., Curtis W., Pawlosky R., King M.T. (2017). Ketone bodies mimic the life span extending properties of caloric restriction. IUBMB Life.

[17] Veech R.L., Chaance B., Kashiwaya Y., Lardy H.A., Cahill G.C. (2001). Ketone bodies, potential therapeutic uses. IUBMB Life.

[18] Veech R.L. (2004). The therapeutic implications of ketone bodies: The effects of ketone bodies in pathological conditions: Ketosis, ketogenic diet, redox states, insulin resistance, and mitochondrial metabolism. Prostaglandins Leukot. Essent. Fat. Acids.

[19] Poffé C., Ramaekers M., Bogaerts S., Hespel P. (2021). Bicarbonate Unlocks the Ergogenic Action of Ketone Monoester Intake in Endurance Exercise. Med. Sci. Sports Exerc..

[20] Poffé C., Ramaekers M., Bogaerts S., Hespel P. (2020). Exogenous ketosis impacts neither performance nor muscle glycogen breakdown in prolonged endurance exercise. J. Appl. Physiol..

[21] Cox P.J., Kirk T., Ashmore T., Willerton K., Evans R., Smith A., Murray A., Stubbs B., West J., McLure S.W. (2016). Nutritional Ketosis Alters Fuel Preference and Thereby Endurance Performance in Athletes. Cell Metab..

[22] Waldman H.S., Basham S.A., Price F.G., Smith J.W., Chander H., Knight A.C., Krings B.M., McAllister M.J. (2018). Exogenous ketone salts do not improve cognitive responses after a high-intensity exercise protocol in healthy college-aged males. Appl. Physiol. Nutr. Metab..

[23] Mujica-Parodi L.R., Amgalan A., Sultan S.F., Antal B., Sun X., Skiena S., Lithen A., Adra N., Ratai E.-M., Weistuch C. (2020). Diet modulates brain network stability, a biomarker for brain aging, in young adults. Proc. Natl. Acad. Sci. USA.

[24] Evans M., Egan B. (2018). Intermittent Running and Cognitive Performance after Ketone Ester Ingestion. Med. Sci. Sports Exerc..

[25] Avgerinos I.K., Egan J.M., Mattson M.P., Kapogiannis D. (2020). Medium Chain Triglycerides induce mild ketosis and may improve cognition in Alzheimer’s disease. A systematic review and meta-analysis of human studies. Ageing Res. Rev..

[26] Walsh J.J., Neudorf H., Little J.P. (2021). 14-Day Ketone Supplementation Lowers Glucose and Improves Vascular Function in Obesity: A Randomized Crossover Trial. J. Clin. Endocrinol. Metab..

[27] Myette-Côté É., Neudorf H., Rafiei H., Clarke K., Little J.P. (2018). Prior ingestion of exogenous ketone monoester attenuates the glycaemic response to an oral glucose tolerance test in healthy young individuals. J. Physiol..

[28] Myette-Côté É., Caldwell H.G., Ainslie P.N., Clarke K., Little J.P. (2019). A ketone monoester drink reduces the glycemic response to an oral glucose challenge in individuals with obesity: A randomized trial. Am. J. Clin. Nutr..

[29] Soto-Mota A., Norwitz N.G., Clarke K. (2020). Why a d-beta-hydroxybutyrate monoester?. Biochem. Soc. Trans..

[30] Soto-Mota A., Vansant H., Evans R.D., Clarke K. (2019). Safety and tolerability of sustained exogenous ketosis using ketone monoester drinks for 28 days in healthy adults. Regul. Toxicol. Pharmacol..

[31] Stefan M., Sharp M., Gheith R., Lowery R., Wilson J. (2020). The Effects of Exogenous Beta-Hydroxybutyrate Supplementation on Metrics of Safety and Health. Int. J. Nutr. Food Sci..

[32] Fortier M., Castellano C., St-Pierre V., Myette-Côté É., Langlois F., Roy M., Morin M., Bocti C., Fulop T., Godin J. (2021). A ketogenic drink improves cognition in mild cognitive impairment: Results of a 6-month RCT. Alzheimer’s Dement..

[33] Stubbs B.J., Cox P.J., Kirk T., Evans R.D., Clarke K. (2019). Gastrointestinal Effects of Exogenous Ketone Drinks are Infrequent, Mild, and Vary According to Ketone Compound and Dose. Int. J. Sport Nutr. Exerc. Metab..

[34] Jeukendrup E.A., Thielen J.J., Wagenmakers A.J., Brouns F., Saris W.H. (1998). Effect of medium-chain triacylglycerol and carbohydrate ingestion during exercise on substrate utilization and subsequent cycling performance. Am. J. Clin. Nutr..

[35] Wibisono C., Rowe N., Beavis E., Kepreotes H., Mackie F.E., Lawson J.A., Cardamone M. (2015). Ten-Year Single-Center Experience of the Ketogenic Diet: Factors Influencing Efficacy, Tolerability, and Compliance. J. Pediatr..

[36] Stubbs B.J., Blade T., Mills S., Thomas J., Yufei X., Nelson F.R., Higley N., Nikiforov A.I., Rhiner M.O., Verdin E. (2021). In vitro stability and in vivo pharmacokinetics of the novel ketogenic ester, bis hexanoyl (R)-1,3-butanediol. Food Chem. Toxicol..

[37] Declaration of Helsinki (2008). Ethical Principles for Medical Research Involving Human Subjects. Adopted by the 18th WMA General Assembly, Helsinki, Finland, June 1964, and Amended by the: 59th WMA General Assembly, Seoul, October 2008.

[38] Boler B.M.V., Serao M.C.R., Bauer L.L., Staeger M.A., Boileau T.W., Swanson K., Fahey G.C. (2011). Digestive physiological outcomes related to polydextrose and soluble maize fibre consumption by healthy adult men. Br. J. Nutr..

[39] Maki K.C., Rains T.M., Kelley K.M., Cook C.M., Schild A.L., Gietl E. (2012). Fibermalt is well tolerated in healthy men and women at intakes up to 60 g/d: A randomized, double-blind, crossover trial. Int. J. Food Sci. Nutr..

[40] Martin C.S., Earleywine M., Musty R.E., Perrine M.W., Swift R.M. (1993). Development and Validation of the Biphasic Alcohol Effects Scale. Alcohol. Clin. Exp. Res..

[41] Rueger S.Y., King A.C. (2012). Validation of the Brief Biphasic Alcohol Effects Scale (B-BAES). Alcohol. Clin. Exp. Res..

[42] Peterson J.I., Young D.S. (1968). Evaluation of the hexokinase-glucose-6-phosphate dehydrogenase method of determination of glucose in urine. Anal. Biochem..

[43] Friedewald W.T., Levy R.I., Fredrickson D.S. (1972). Estimation of the Concentration of Low-Density Lipoprotein Cholesterol in Plasma, Without Use of the Preparative Ultracentrifuge. Clin. Chem..

[44] Ivy J.L., Costill D.L., Fink W.J., Maglischo E. (1980). Contribution of Medium and Long Chain Triglyceride Intake to Energy Metabolism during Prolonged Exercise. Int. J. Sports Med..

[45] Shah N., Limketkai B. (2017). The Use of Medium-Chain Triglycerides in Gastrointestinal Disorders. Pract. Gastroenterol..

[46] Liu Y.-M., Wang H.-S. (2013). Medium-chain Triglyceride Ketogenic Diet, An Effective Treatment for Drug-resistant Epilepsy and A Comparison with Other Ketogenic Diets. Biomed. J..

[47] Ohnuma T., Toda A., Kimoto A., Takebayashi Y., Higashiyama R., Tagata Y., Ito M., Ota T., Shibata N., Arai H. (2016). Benefits of use, and tolerance of, medium-chain triglyceride medical food in the management of Japanese patients with Alzheimer’s disease: A prospective, open-label pilot study. Clin. Interv. Aging.

[48] Liu Y.-M.C. (2008). Medium-chain triglyceride (MCT) ketogenic therapy. Epilepsia.

[49] Goedecke J.H., Clark V.R., Noakes T.D., Lambert E. (2005). The Effects of Medium-Chain Triacylglycerol and Carbohydrate Ingestion on Ultra-Endurance Exercise Performance. Int. J. Sport Nutr. Exerc. Metab..

[50] Stubbs B., Cox P.J., Evans R.D., Santer P., Miller J., Faull O.K., Magor-Elliott S., Hiyama S., Stirling M., Clarke K. (2017). On the Metabolism of Exogenous Ketones in Humans. Front. Physiol..

[51] Rittig N., Svart M., Thomsen H.H., Vestergaard E.T., Rehfeld J.F., Hartmann B., Holst J.J., Johannsen M., Moller N., Jessen N. (2020). Oral D/L-3-Hydroxybutyrate Stimulates Cholecystokinin and Insulin Secretion and Slows Gastric Emptying in Healthy Males. J. Clin. Endocrinol. Metab..

[52] Fischer T., Och U., Klawon I., Och T., Grüneberg M., Fobker M., Bordewick-Dell U., Marquardt T. (2018). Effect of a Sodium and Calcium DL-β-Hydroxybutyrate Salt in Healthy Adults. J. Nutr. Metab..

[53] Evans M., Patchett E., Nally R., Kearns R., Larney M., Egan B. (2018). Effect of acute ingestion of β-hydroxybutyrate salts on the response to graded exercise in trained cyclists. Eur. J. Sport Sci..

[54] Chander H., McAllister M.J., Holland A.M., Waldman H.S., Krings B.M., Swain J.C., Turner A.J., Basham S.A., Smith J.W., Knight A.C. (2019). Effects of 7-Day Ketone Ingestion and a Physiological Workload on Postural Stability, Cognitive, and Muscular Exertion Measures in Professional Firefighters. Safety.

[55] Leckey J.J., Ross M.L., Quod M., Hawley J.A., Burke L.M. (2017). Ketone Diester Ingestion Impairs Time-Trial Performance in Professional Cyclists. Front. Physiol..

[56] David M.S., Merien F., Braakhuis A., Plews D., Laursen P., Dulson D.K. (2019). The Effect of 1,3-Butanediol on Cycling Time-Trial Performance. Int. J. Sport Nutr. Exerc. Metab..

[57] Vandoorne T., De Smet S., Ramaekers M., Van Thienen R., De Bock K., Clarke K., Hespel P. (2017). Intake of a Ketone Ester Drink during Recovery from Exercise Promotes mTORC1 Signaling but Not Glycogen Resynthesis in Human Muscle. Front. Physiol..

[58] Evans M., Mcswiney F.T., Brady A.J., Egan B. (2019). No Benefit of Ingestion of a Ketone Monoester Supplement on 10-km Running Performance. Med. Sci. Sports Exerc..

[59] Clarke K., Tchabanenko K., Pawlosky R., Carter E., King M.T., Musa-Veloso K., Ho M., Roberts A., Robertson J., VanItallie T.B. (2012). Kinetics, safety and tolerability of (R)-3-hydroxybutyl (R)-3-hydroxybutyrate in healthy adult subjects. Regul. Toxicol. Pharmacol..

[60] Schrieks I.C., Stafleu A., Kallen V.L., Grootjen M., Witkamp R., Hendriks H.F.J. (2014). The Biphasic Effects of Moderate Alcohol Consumption with a Meal on Ambiance-Induced Mood and Autonomic Nervous System Balance: A Randomized Crossover Trial. PLoS ONE.

[61] Fridberg D.J., Rueger S.Y., Smith P., King A.C. (2017). Association of Anticipated and Laboratory-Derived Alcohol Stimulation, Sedation, and Reward. Alcohol. Clin. Exp. Res..

[62] Greaves G., Xiang R., Rafiei H., Malas A., Little J.P. (2020). Prior ingestion of a ketone monoester supplement reduces postprandial glycemic responses in young healthy-weight individuals. Appl. Physiol. Nutr. Metab..

