# Supplementary material for: Tolerability and Safety of a Novel Ketogenic Ester, Bis-Hexanoyl (R)-1,3-Butanediol: A Randomized Controlled Trial in Healthy Adults
Source: Nutrients. 2021 Jun 16;13(6):2066. doi: 10.3390/nu13062066 (PMC8234448; doi:10.3390/nu13062066)

Supplemental Information:

Full inclusion and exclusion criteria:

Inclusion Criteria

1. Subject is male or female, 18-65 years of age, inclusive at Visit 1 (Day -7).
2. Subject has a BMI 18.5-34.9 kg/m^2 (inclusive) at Visit 1 (Day -7).
3. Subject is willing and able to comply with all study procedures including consumption of breakfast and lunch daily, maintenance of habitual dietary intake, exercise and medication and supplement use, blood draws and the following prior to test visits: fasting (≥10 h; water only), no alcohol (≥10 h), no cannabis products (≥10 h) and no exercise (≥10 h).
4. Subject has internet access via computer, phone, or other device and is able to maintain internet access throughout the trial in order to complete online daily questionnaires.
5. Subject has no health conditions that would prevent him from fulfilling the study requirements as judged by the Clinical Investigator on the basis of medical history and routine laboratory test results.
6. Subject understands the study procedures and signs forms providing informed consent to participate in the study and authorizes the release of relevant protected health information to the Clinical Investigator.

Exclusion Criteria

1. Subject has an abnormal laboratory test result(s) of clinical importance at Visit 1 (Day -7), at the discretion of the Clinical Investigator. One re-test will be allowed on a separate day prior to Visit 2 (Day 0), for subjects with abnormal laboratory test results.
2. Subject has a history or presence of uncontrolled and/or clinically important pulmonary, cardiac, hepatic, renal, endocrine (including type 1 and 2 diabetes), hematologic, immunologic, neurologic (e.g., Alzheimer's or Parkinson's diseases), psychiatric (including unstable depression and/or anxiety disorders) or biliary disorders.
3. Subject has a clinically important gastrointestinal (GI) condition that would potentially interfere with the evaluation of the study beverage [e.g., inflammatory bowel disease, irritable bowel syndrome, chronic constipation, severe constipation (in the opinion of the Clinical Investigator), history of frequent diarrhea, history of surgery for weight loss, gastroparesis, systemic disease that might affect gut motility according to the Investigator, medication managed reflux and/or clinically important lactose intolerance].
4. Subject is a female who is pregnant, planning to be pregnant during the study period, lactating, or is of childbearing potential and is unwilling to commit to the use of a medically approved form of contraception throughout the study period. The method of contraception must be recorded.
5. Subject is a current user of tobacco, smoking products (including, but not limited to cigarettes, cigars, chewing tobacco, e-cigarettes), and nicotine products (e.g., nicotine gum and/or nicotine patches) within 6 months of Visit 1 (Day -7) at the equivalent of >5 cigarettes per day.
6. Subject has a history of or strong potential for alcohol or substance abuse.
7. Subject is consistently using prescriptive or over-the counter medications where alcohol is a contraindication at the discretion of the Investigator.

8. Subject has a known allergy, intolerance, or sensitivity to any of the ingredients in the study beverages, including soy and milk protein, wheat, shellfish, fin fish, eggs, tree nuts or peanuts (production facility handles nuts).
9. Subject has uncontrolled hypertension (systolic blood pressure ≥140 mm Hg or diastolic blood pressure ≥90 mm Hg) as defined by the blood pressure measured at Visit 1 (Day -7). One re-test will be allowed on a separate day before Visit 2 (Day 0), for subjects with abnormal blood pressure.
10. Subject has a history or presence of cancer in the prior two years, except for non-melanoma skin cancer.
11. Subject has experienced any major trauma or any other surgical event within three months of Visit 1 (Day -7).
12. Subject has recently used antibiotics (within 30 days of visit 1, Day -7).
13. Subject has extreme dietary habits (e.g., intermittent fasting or time restricted eating, Atkins diet, vegan, very high protein/low carbohydrate or has used weight-loss medications (including over-the-counter medications and/or supplements) or programs within 30 days of Visit 1 (Day -7).
14. Subject has used medications (over-the-counter or prescription) known to influence gastrointestinal function including, but not limited to, opioids, weight loss medications, anti-diarrheals, and anti-spasmodics) within 30 days of Visit 1 (Day -7).
15. Subject has unstable use of constipation medications, supplements or beverages (over-the-counter or prescription), including but not limited, fiber, pre- and probiotics, laxatives, enemas, suppositories within 2 months of Visit 1 (Day -7).
16. Subject consistently uses anti-inflammatory medications (>5 times/week), including over-the-counter or prescription steroid and non-steroidal anti-inflammatory drugs, within 30 days of Visit 1 (Day -7).
17. Subject has used ketone supplements (ketone salts or esters, and medium chain triglycerides [MCT]) within 30 days of Visit 1 (Day -7).
18. Subject has unstable use of thyroid, antihypertensive, antidepressant, or statin medications within 6 months of Visit 1 (Day -7).
19. Individual has a condition the Clinical Investigator believes would interfere with his ability to provide informed consent, comply with the study protocol, which might confound the interpretation of the study results, or put the subject at undue risk.
20. Subject works nights or shifts that means it is not possible to maintain a consistent meal schedule during the study.

Excluded Medications/Supplements/Products

Unstable use of any thyroid hormone therapy, statins, antihypertensives, and antidepressants within 6 months of Visit 1 (Day -7) or constipation medications or supplements within 2 months of Visit 1 (Day-7) is not allowed and throughout the study period. Additionally, use of any antibiotic therapy is not permitted within 30 days of Visit 1 (Day -7) and throughout the study period. Subjects should not use opioids, weight loss medications, anti-diarrheals, anti-spasmodics, ketone supplements (including MCT oil), or other medications (over-the-counter or prescription) or dietary supplements known to alter gastrointestinal function within 30 days of Visit 1 (Day -7) and throughout the study period, with the exception of stable use of constipation medications and supplements. Unstable use of constipation medications or supplements (prescription or over-the-

counter) is not permitted within 2 months of Visit 1 (Day -7). Additionally, regular use (>5 times/week) of anti-inflammatory medications (prescribed or over-the-counter) is also not allowed 30 days prior to Visit 1 (Day -7). Occasional use of over-the-counter anti-inflammatory medications are allowed if taken <5 times/week. Should a subject require any of these medications or supplements, the study staff should consult with the Project Manager to discuss the subject's continued participation in the trial.

Supplemental Table S1:

Clinical chemistry measures at clinic visits during 28-day study of healthy adults consuming up to 25 g/day of BH-BD or placebo.

		BH-BD		Placebo	
Test	Time point (day)	Mean (SD)	p-value[1]	Mean (SD)	p-value[1]
A/G RATIO	0	1.2 (0.2)	-	1.3 (0.2)	-
	7	1.2 (0.2)	0.75	1.3 (0.2)	0.32
	14	1.2 (0.1)	0.62	1.2 (0.2)	0.26
	28	1.2 (0.2)	0.27	1.3 (0.2)	0.54
ALBUMIN (g/dL)	0	3.9 (0.3)	-	3.9 (0.3)	-
	7	3.9 (0.2)	0.56	3.9 (0.3)	0.58
	14	3.8 (0.2)	0.19	3.9 (0.4)	0.54
	28	3.8 (0.3)	0.23	3.9 (0.4)	0.26
ALKALINE PHOSPHATASE (U/L)	0	78.1 (22.8)	-	69.4 (19.2)	-
	7	78.2 (22.7)	0.98	69.8 (19.0)	0.65
	14	76.8 (21.8)	0.54	71.3 (17.5)	0.19
	28	76.1 (21.3)	0.18	68.8 (16.4)	0.27
ALT (U/L)	0	38.6 (25.3)	-	31.3 (16.1)	-
	7	43.0 (38.2)	0.31	29.8 (11.4)	0.33
	14	37.5 (29.8)	0.032	30.6 (14.3)	0.81
	28	34.0 (21.0)	0.008	29.8 (13.0)	0.18
ANION GAP (mmol/L)	0	4.6 (1.8)	-	4.1 (1.6)	-
	7	4.5 (1.8)	0.91	4.5 (1.6)	0.30
	14	4.9 (1.3)	0.37	4.6 (1.4)	0.13
	28	4.1 (2.1)	0.30	4.7 (1.9)	0.25
AST (U/L)[2]	0	21.2 (7.8)	-	19.2 (7.2)	-
	7	30.1 (62.8)	0.14	19.4 (6.3)	0.94
	14	20.6 (14.5)	0.055	19.0 (11.0)	0.20
	28	18.0 (7.2)	<0.001	17.8 (5.6)	0.054
BILIRUBIN, TOTAL[3] (mg/dL)	0	0.6 (0.3)	-	0.7 (0.3)	-
	7	0.6 (0.3)	1.00	0.7 (0.4)	0.55
	14	0.5 (0.2)	0.20	0.7 (0.5)	0.87
	28	0.5 (0.2)	0.009	0.8 (0.9)	0.49
BUN (mg/dL)	0	14.8 (3.7)	-	12.9 (3.6)	-
	7	15.3 (3.9)	0.35	13.5 (3.8)	0.33
	14	14.6 (3.7)	0.76	13.9 (3.0)	0.028

		BH-BD		Placebo	
Test	Time point (day)	Mean (SD)	p-value[1]	Mean (SD)	p-value[1]
	28	14.6 (3.8)	0.74	13.9 (3.2)	0.068
BUN/CREA RATIO	0	16.3 (3.9)	-	15.1 (5.5)	-
	7	17.4 (4.8)	0.11	15.3 (5.1)	0.42
	14	17.0 (5.6)	0.37	16.5 (5.5)	0.008
	28	17.4 (5.3)	0.26	16.5 (5.0)	0.023
CALCIUM (mg/dL)[4]	0	9.3 (0.5)	-	9.2 (0.4)	-
	7	9.3 (0.5)	0.27	9.3 (0.3)	0.088
	14	9.3 (0.5)	0.40	9.4 (0.4)	0.010
	28	9.2 (0.5)	0.018	9.3 (0.3)	0.28
CALCULATED OSMOLALITY (mOsm/kg)	0	289.0 (3.7)	-	288.8 (4.8)	-
	7	288.1 (5.1)	0.34	288.4 (4.5)	0.66
	14	289.0 (4.5)	0.65	287.6 (4.4)	0.16
	28	289.3 (4.3)	0.50	288.8 (3.8)	0.91
CHLORIDE (mmol/L)	0	105.7 (2.1)	-	106.7 (2.7)	-
	7	105.5 (2.4)	0.39	106.5 (2.2)	0.53
	14	106.0 (1.9)	0.63	105.9 (2.5)	0.044
	28	106.0 (2.5)	0.52	106.1 (2.4)	0.19
CO_2 (mmol/L)	0	28.9 (2.2)	-	28.7 (2.0)	-
	7	28.8 (2.2)	0.58	28.3 (2.1)	0.30
	14	28.5 (1.8)	0.44	28.4 (2.1)	0.65
	28	29.4 (2.3)	0.28	28.6 (2.0)	0.84
CREATININE (mg/dL)[5]	0	0.9 (0.2)	-	0.9 (0.2)	-
	7	0.9 (0.2)	0.15	0.9 (0.2)	0.29
	14	0.9 (0.2)	0.044	0.9 (0.2)	0.58
	28	0.9 (0.2)	0.006	0.9 (0.2)	0.30
GLOBULIN (g/dL)[6]	0	3.3 (0.3)	-	3.2 (0.4)	-
	7	3.2 (0.4)	0.33	3.2 (0.3)	0.87
	14	3.2 (0.3)	0.77	3.2 (0.3)	0.34
	28	3.1 (0.3)	0.019	3.1 (0.4)	0.51
GLUCOSE (mg/dL)[7]	0	93.4 (7.8)	-	91.6 (10.5)	-
	7	91.0 (9.0)	0.021	88.5 (7.5)	0.039
	14	92.4 (11.6)	0.17	87.1 (8.2)	0.001
	28	92.0 (11.4)	0.19	88.6 (7.7)	0.043
POTASSIUM (mmol/L)	0	4.6 (0.6)	-	4.6 (0.8)	-
	7	4.6 (0.7)	0.95	4.7 (0.8)	0.49
	14	4.4 (0.3)	0.53	4.5 (0.7)	0.97

		BH-BD		Placebo	
**Test**	**Time point (day)**	**Mean (SD)**	**p-value[1]**	**Mean (SD)**	**p-value[1]**
	28	4.4 (0.3)	0.32	4.4 (0.3)	0.18
SODIUM (mmol/L)	0	139.3 (1.8)	-	139.5 (2.1)	-
	7	138.8 (2.4)	0.25	139.3 (2.1)	0.70
	14	139.4 (1.8)	0.56	139.0 (2.1)	0.11
	28	139.5 (2.0)	0.52	139.4 (1.8)	0.77
TOTAL PROTEIN (g/dL)[8]	0	7.1 (0.4)	-	7.0 (0.5)	-
	7	7.1 (0.5)	0.28	7.0 (0.4)	0.89
	14	7.1 (0.4)	0.24	7.1 (0.3)	0.28
	28	7.0 (0.4)	0.014	7.0 (0.5)	0.89

Abbreviations: AST, Aspartate Aminotransferase; ALT, Alanine Aminotransferase; A/G, Albumin/Globulin ratio; BH-BD, bis-hexanoyl (R)-1,3-butanediol, BHB, beta-hydroxybutyrate; BUN, Blood Urea Nitrogen; CREA, Creatinine; SD, standard deviation.

[1]P-values represent within group changes from baseline (day 0) and were compared using Wilcoxon sign rank test. No corrections for multiple comparisons were made.

[2] AST, normal range = 15 – 37 U/L. The mean AST fell during the study in the BH-BD group; low AST was not considered to be a safety concern.

[3] Bilirubin, total, normal range = 0.1 -2.0 mg/dL. The small change in bilirubin in the BH-BD group during the study was statistically significant but not clinically meaningful as values were within the normal range.

[4] Calcium, normal range = 8.5 – 10.1 mg/dL. The small change in calcium in the BH-BD group during the study was statistically significant but not clinically meaningful as values were within the normal range.

[5] Creatinine, normal range = female: 0.55 – 1.02 mg/dL; male = 0.70 – 1.30 mg/dL. The small change in creatinine in the BH-BD group during the study was statistically significant but not clinically meaningful as values were within the normal ranges.

[6] Globulin, normal range = 2.8-4.4 g/dL. The small change in globulin in the BH-BD group during the study was statistically significant but not clinically meaningful as values were within the normal ranges.

[7]Glucose, normal range =

[8] Total protein, normal range = 6.4 – 8.2 g/dL. The small change in total protein in the BH-BD group during the study was statistically significant but not clinically meaningful as values were within the normal ranges.

Supplemental Table S2: Haematology measures at clinic visits during 28-day study of healthy adults consuming up to 25 g/day of BH-BD or placebo.

		BH-BD		Placebo	
Test	Time point (day)	Mean (SD)	p-value[1]	Mean (SD)	p-value[1]
BASOPHIL % (%)	0	0.8 (0.4)	-	0.8 (0.4)	-
	7	0.8 (0.3)	0.59	0.8 (0.4)	0.72
	14	0.8 (0.3)	0.88	0.7 (0.3)	0.65
	28	0.8 (0.3)	0.38	0.7 (0.3)	0.13
BASOPHIL ABSOLUTE (x10^3 µL)	0	0.05 (0.03)	-	0.04 (0.02)	-
	7	0.05 (0.02)	0.76	0.04 (0.02)	1.00
	14	0.05 (0.02)	0.31	0.04 (0.02)	0.90
	28	0.05 (0.02)	0.64	0.04 (0.02)	0.99
EOSINOPHIL % (%)	0	2.7 (2.2)	-	3.6 (2.4)	-
	7	2.6 (2.2)	0.43	3.4 (2.3)	0.22
	14	2.8 (2.3)	0.15	3.3 (2.3)	0.12
	28	2.9 (2.1)	0.067	3.1 (2.0)	0.068
EOSINOPHIL ABSOLUTE (x10^3 µL)	0	0.2 (0.2)	-	0.2 (0.2)	-
	7	0.2 (0.2)	0.058	0.2 (0.2)	0.080
	14	0.2 (0.2)	0.93	0.2 (0.2)	0.49
	28	0.2 (0.1)	0.82	0.2 (0.2)	0.40
HEMATOCRIT (%)	0	43.0 (3.8)	-	42.7 (4.3)	-
	7	42.8 (4.0)	0.22	42.7 (4.1)	0.86
	14	42.6 (3.6)	0.079	42.9 (3.9)	0.82
	28	42.5 (3.6)	0.12	42.5 (3.8)	0.48
HEMOGLOBIN (g/dL)[2]	0	14.1 (1.4)	-	14.0 (1.5)	-
	7	14.0 (1.6)	0.17	14.0 (1.5)	0.90
	14	13.9 (1.5)	0.042	14.0 (1.4)	0.87
	28	13.9 (1.5)	0.11	14.0 (1.5)	0.80
IMMATURE GRANULOCYTE % (%)	0	0.2 (0.2)	-	0.3 (0.2)	-
	7	0.3 (0.4)	0.12	0.3 (0.2)	0.95
	14	0.2 (0.2)	0.74	0.4 (0.6)	0.16
	28	0.3 (0.2)	0.14	0.2 (0.2)	0.29
IMMATURE GRANULOCYTE ABSOLUTE (x10^3 µL)[3]	0	0.02 (0.02)	-	0.02 (0.01)	-
	7	0.02 (0.03)	0.032	0.02 (0.01)	0.61
	14	0.02 (0.01)	0.89	0.02 (0.04)	0.16
	28	0.02 (0.02)	0.56	0.01 (0.01)	0.99

		BH-BD		Placebo	
Test	Time point (day)	Mean (SD)	p-value[1]	Mean (SD)	p-value[1]
LYMPHOCYTE % (%)	0	33.0 (7.2)	-	35.5 (8.5)	-
	7	32.7 (9.7)	0.86	35.7 (6.0)	0.90
	14	33.4 (7.9)	0.46	36.2 (7.0)	0.78
	28	34.3 (7.7)	0.088	36.1 (8.6)	0.55
LYMPHOCYTE ABSOLUTE (x10^3 µL)	0	2.2 (0.6)	-	2.0 (0.6)	-
	7	2.1 (0.7)	0.071	2.0 (0.5)	0.47
	14	2.1 (0.7)	0.37	2.1 (0.6)	0.016
	28	2.2 (0.6)	0.91	2.2 (0.6)	0.012
MCH (pg)	0	29.9 (1.6)	-	29.1 (1.8)	-
	7	29.8 (1.6)	0.30	29.0 (1.9)	0.43
	14	29.8 (1.6)	0.14	29.0 (1.8)	0.20
	28	29.8 (1.7)	0.16	29.1 (1.8)	0.61
MCHC (g/dL)	0	32.7 (0.8)	-	32.7 (1.0)	-
	7	32.7 (1.0)	0.92	32.6 (0.9)	0.60
	14	32.6 (0.9)	0.22	32.7 (0.9)	0.76
	28	32.7 (1.1)	0.72	32.8 (1.1)	0.55
MCV (fL)	0	91.4 (4.0)	-	88.9 (4.7)	-
	7	91.2 (4.0)	0.23	88.9 (4.7)	0.80
	14	91.4 (3.8)	0.96	88.6 (4.5)	0.24
	28	91.2 (3.6)	0.35	88.6 (4.5)	0.054
MONOCYTE % (%)	0	8.6 (1.6)	-	8.4 (2.1)	-
	7	8.5 (2.0)	0.64	8.7 (1.9)	0.27
	14	8.6 (2.0)	0.77	8.3 (1.7)	0.73
	28	9.4 (2.2)	0.063	8.7 (1.9)	0.074
MONOCYTE ABSOLUTE (x10^3 µL)	0	0.6 (0.2)	-	0.5 (0.1)	-
	7	0.5 (0.2)	0.067	0.5 (0.1)	0.13
	14	0.5 (0.2)	0.43	0.5 (0.2)	0.20
	28	0.6 (0.2)	0.43	0.5 (0.1)	<.001
NEUTROPHIL % (%)	0	54.7 (7.4)	-	51.4 (8.1)	-
	7	55.1 (11.2)	0.93	51.2 (6.1)	0.73
	14	54.2 (8.7)	0.41	51.2 (6.2)	0.96
	28	52.3 (7.1)	0.003	51.1 (8.6)	0.67
PLATELETS (10^3 µL)	0	280.6 (52.4)	-	255.8 (50.7)	-
	7	278.0 (58.1)	0.67	250.3 (49.7)	0.13
	14	288.0 (53.3)	0.47	260.3 (52.4)	0.70

		BH-BD		Placebo	
**Test**	**Time point (day)**	**Mean (SD)**	**p-value[1]**	**Mean (SD)**	**p-value[1]**
	28	286.9 (54.0)	0.28	253.0 (51.6)	0.37
RDW (%)[4]	0	12.7 (0.7)	-	13.0 (1.1)	-
	7	12.6 (0.7)	0.058	12.9 (1.1)	0.68
	14	12.6 (0.7)	0.044	13.0 (1.2)	0.92
	28	12.7 (0.7)	0.62	13.0 (1.1)	0.74
RDW-SD (fL)	0	42.6 (2.4)	-	41.9 (3.4)	-
	7	42.3 (2.7)	0.14	42.0 (3.2)	0.61
	14	42.3 (2.5)	0.22	41.8 (3.4)	0.38
	28	42.4 (2.6)	0.44	41.8 (3.0)	0.50
RED BLOOD COUNT ($x10^6$ µL)	0	4.7 (0.4)	-	4.8 (0.6)	-
	7	4.7 (0.4)	0.36	4.8 (0.6)	0.78
	14	4.7 (0.4)	0.14	4.9 (0.6)	0.65
	28	4.7 (0.4)	0.18	4.8 (0.6)	0.89
WBC ($x10^3$ µL)	0	6.7 (1.7)	-	5.7 (1.5)	-
	7	6.4 (1.4)	0.10	5.6 (1.4)	0.76
	14	6.2 (1.3)	0.20	5.9 (1.7)	0.10
	28	6.3 (1.4)	0.12	6.2 (1.5)	<.001

Abbreviations: BH-BD, bis-hexanoyl (R)-1,3-butanediol, B-BAES, Brief Biphasic Alcohol Effect Scale; BHB, beta-hydroxybutyrate; BTQ, Beverage Tolerability Questionnaire; N/A, not applicable (as all values were in the normal range); SD, standard deviation (MCH, Mean Corpuscular Hemoglobin; MCHC, Mean Corpuscular Hemoglobin Concentration, MCV, Mean Corpuscular Volume; RDW, Red cell distribution width; RDW-SD, Red cell distribution width (standard deviation); WBC, White Blood Cells.

[1] P-values represent within group changes from baseline (day 0) and were compared using Wilcoxon sign rank test.) No correction for multiple comparisons were performed.

[2] Haemoglobin, normal range = 12.0 – 17.5 g/dL. The small change in haemoglobin in the BH-BD group during the study was statistically significant but not clinically meaningful as values were within the normal range supplied by Elmhurst Memorial Lab (IL).

[3] Immature granulocytes, normal range = 0.00 – 1.00 k/UL. The small change in immature granulocytes in the BH-BD group during the study was statistically significant but not clinically meaningful as values were within the normal ranges supplied by Elmhurst Memorial Lab (IL).

[4] RDW, normal range = 11 – 15 %. The small change in RDW in the BH-BD group during the study was statistically significant but not clinically meaningful as values were within the normal ranges supplied by Elmhurst Memorial Lab (IL).

Supplemental Table S3: Blood lipid measures at clinic visits during 28-day study of healthy adults consuming up to 25 g/day of BH-BD or placebo.

		BH-BD		Placebo	
**Test**	**Time point (day)**	**mean (sd)**	**p-value[1]**	**mean (sd)**	**p-value[1]**
APOLIPOPROTEIN, B (mg/dL)	0	94.2 (22.0)	-	82.3 (25.8)	-
	7	94.5 (21.3)	0.88	79.9 (24.6)	0.40
	14	94.4 (25.7)	0.94	78.9 (22.4)	0.18
	28	93.2 (22.1)	0.66	79.3 (20.7)	0.20
CHOLESTEROL, TOTAL (mg/dL)	0	200.1 (38.3)	-	187.2 (47.9)	-
	7	193.9 (39.5)	0.21	180.5 (37.4)	0.14
	14	197.3 (44.0)	0.85	180.2 (40.0)	0.30
	28	197.8 (40.7)	0.68	179.8 (39.7)	0.037
HDL CHOLESTEROL (mg/dL)	0	57.4 (20.2)	-	63.0 (32.1)	-
	7	57.7 (21.9)	0.83	63.0 (28.6)	0.56
	14	55.4 (21.1)	0.049	63.0 (28.9)	0.93
	28	55.1 (19.5)	0.15	60.9 (26.0)	0.39
LDL CHOLESTEROL (mg/dL)	0	117.2 (31.5)	-	104.6 (34.0)	-
	7	111.0 (30.0)	0.092	97.2 (28.6)	0.081
	14	118.9 (37.7)	0.60	97.5 (32.7)	0.099
	28	116.5 (31.2)	0.77	99.7 (29.9)	0.16
NON-HDL CHOLESTEROL (mg/dL)	0	142.7 (31.4)	-	124.1 (37.6)	-
	7	136.2 (32.8)	0.092	117.5 (32.5)	0.13
	14	141.9 (40.6)	0.97	117.3 (35.7)	0.18
	28	142.8 (35.8)	0.85	118.9 (32.2)	0.10
TRIGLYCERIDES (mg/dL)	0	127.5 (70.7)	-	98.1 (42.6)	-
	7	125.9 (63.9)	0.99	101.6 (53.1)	0.44
	14	115.3 (55.6)	0.18	98.9 (39.0)	0.77
	28	131.8 (69.6)	0.92	96.3 (39.1)	0.89
VLDL CHOLESTEROL (mg/dL)	0	25.5 (14.2)	-	19.6 (8.5)	-
	7	25.1 (12.8)	0.96	20.3 (10.6)	0.43
	14	23.0 (11.2)	0.19	19.8 (7.8)	0.72
	28	26.3 (13.9)	0.91	19.2 (7.8)	0.94

Abbreviations: BH-BD, bis-hexanoyl (R)-1,3-butanediol, N/A, not applicable (as all values were in the normal range); SD, standard deviation; HDL, high density lipoprotein; LDL, low density lipoprotein; VLDL, very low-density lipoprotein.

[1] P-values represent within group changes from baseline (day 0) and were compared using Wilcoxon sign rank test. No correction for multiple comparisons were performed.

[2] HDL cholesterol, normal range = 40 – 59 mg/dL. The small change in HDL in the BH-BD group during the study was statistically significant but not clinically meaningful as values were within the normal ranges supplied by Elmhurst Memorial Lab (IL).

Supplemental Table S4: Thyroid hormone measures at clinic visits during 28-day study of healthy adults consuming up to 25 g/day of BH-BD or placebo.

		BH-BD		Placebo	
**Test**	**Time point (day)**	**Mean (SD)**	**p-value[1]**	**Mean (SD)**	**p-value[1]**
T3 TOTAL (ng/dL)	0	111.9 (21.9)	-	114.8 (21.3)	-
	7	110.0 (19.2)	0.94	114.7 (25.0)	0.63
	14	108.7 (16.8)	0.37	115.4 (18.1)	0.47
	28	107.7 (16.8)	0.17	115.8 (21.2)	0.91
THYROXINE (T4) (ug/dL)	0	8.7 (1.6)	-	9.2 (2.2)	-
	7	8.9 (1.3)	0.40	9.3 (1.8)	0.49
	14	8.8 (1.4)	0.87	9.4 (1.6)	0.20
	28	8.5 (1.2)	0.23	9.6 (2.0)	0.057
TSH (mIU/mL)[2]	0	2.1 (1.3)	-	2.0 (1.3)	-
	7	1.8 (0.9)	0.076	1.8 (0.9)	0.40
	14	1.8 (0.8)	0.024	1.8 (0.9)	0.053
	28	1.8 (0.7)	0.32	1.8 (0.8)	0.55

Abbreviations: BH-BD, bis-hexanoyl (R)-1,3-butanediol, SD, standard deviation; TSH, thyroid stimulating hormone.

[1] P-values represent within group changes from baseline (day 0) and were compared using Wilcoxon sign rank test. No correction for multiple comparisons were performed.

[2]TSH normal range = 0.358 – 3.74 mIU/mL. The small change in TSH in the BH-BD group during the study was statistically significant but not clinically meaningful as values were within the normal ranges provided by Elmhurst Memorial Lab (IL).

Supplemental Table S5: Urinalysis measures at clinic visits during 28-day study of healthy adults consuming up to 25 g/day of BH-BD or placebo.

Test	Time point (day)	BH-BD Abnormal, n (%)[1]	BH-BD Normal, n (%)[1]	Placebo Abnormal, n (%)[1]	Placebo Normal, n (%)[1]
BILIRUBIN URINE	0		30 (100.0)		29 (100.0)
	7		30 (100.0)		29 (100.0)
	14		30 (100.0)		29 (100.0)
	28		30 (100.0)		29 (100.0)
BLOOD URINE	0	2 (6.7)	28 (93.3)	2 (6.9)	27 (93.1)
	7	3 (10.0)	27 (90.0)	2 (6.9)	27 (93.1)
	14	6 (20.0)	24 (80.0)	3 (10.3)	26 (89.7)
	28	3 (10.0)	27 (90.0)	3 (10.3)	26 (89.7)
CLARITY URINE	0	6 (20.0)	24 (80.0)	6 (20.7)	23 (79.3)
	7	8 (26.7)	22 (73.3)	4 (13.8)	25 (86.2)
	14	8 (26.7)	22 (73.3)	7 (24.1)	22 (75.9)
	28	9 (30.0)	21 (70.0)	3 (10.3)	26 (89.7)
COLOR URINE	0	5 (16.7)	25 (83.3)	3 (10.3)	26 (89.7)
	7	3 (10.0)	27 (90.0)	3 (10.3)	26 (89.7)
	14	4 (13.3)	26 (86.7)	3 (10.3)	26 (89.7)
	28	3 (10.0)	27 (90.0)	3 (10.3)	26 (89.7)
GLUCOSE URINE (mg/dL)	0		30 (100.0)		29 (100.0)
	7		30 (100.0)		29 (100.0)
	14		30 (100.0)		29 (100.0)
	28		30 (100.0)		29 (100.0)
KETONES URINE (mg/dL)	0		30 (100.0)	1 (3.4)	28 (96.6)
	7	1 (3.3)	29 (96.7)	1 (3.4)	28 (96.6)
	14		30 (100.0)	2 (6.9)	27 (93.1)
	28	1 (3.3)	29 (96.7)	5 (17.2)	24 (82.8)
LEUKOCYTE ESTERASE URINE	0	3 (10.0)	27 (90.0)	3 (10.3)	26 (89.7)
	7	5 (16.7)	25 (83.3)	4 (13.8)	25 (86.2)
	14	2 (6.7)	28 (93.3)	2 (6.9)	27 (93.1)
	28	8 (26.7)	22 (73.3)	3 (10.3)	26 (89.7)
NITRITE URINE	0		30 (100.0)		29 (100.0)
	7		30 (100.0)		29 (100.0)
	14		30 (100.0)		29 (100.0)
	28		30 (100.0)		29 (100.0)
PH URINE	0		30 (100.0)		29 (100.0)
	7		30 (100.0)		29 (100.0)
	14		30 (100.0)		29 (100.0)
	28		30 (100.0)		29 (100.0)
PROTEIN URINE (mg/dL)	0	1 (3.3)	29 (96.7)	1 (3.4)	28 (96.6)
	7	1 (3.3)	29 (96.7)	2 (6.9)	27 (93.1)

Test	Time point (day)	BH-BD Abnormal, n (%)[1]	BH-BD Normal, n (%)[1]	Placebo Abnormal, n (%)[1]	Placebo Normal, n (%)[1]
	14		30 (100.0)	3 (10.3)	26 (89.7)
	28	2 (6.7)	28 (93.3)	5 (17.2)	24 (82.8)
SPEC GRAVITY	0		30 (100.0)		29 (100.0)
	7		30 (100.0)		29 (100.0)
	14		30 (100.0)		29 (100.0)
	28		30 (100.0)		29 (100.0)
UROBILINOGEN URINE	0		30 (100.0)		29 (100.0)
	7		30 (100.0)		29 (100.0)
	14		30 (100.0)	1 (3.4)	28 (96.6)
	28		30 (100.0)	1 (3.4)	28 (96.6)

Abbreviations: BH-BD, bis-hexanoyl (R)-1,3-butanediol.

[1]Normal ranges were supplied by Elmhurst Memorial Reference Laboratory (Elmhurst, IL).

CONSORT Diagram

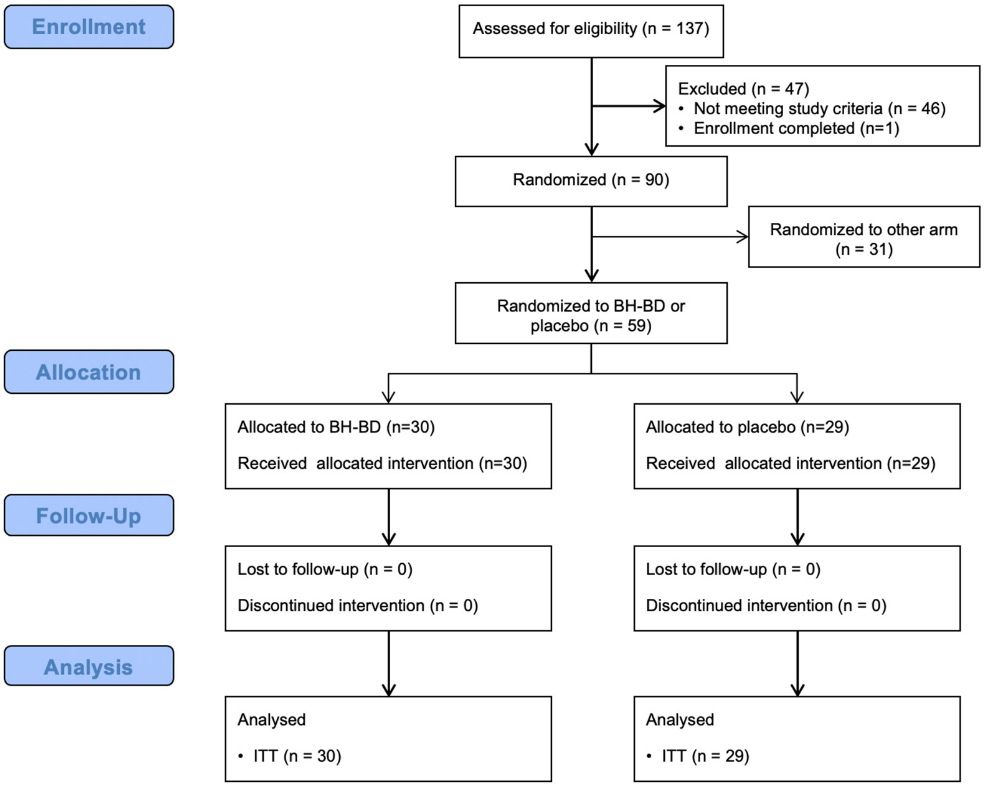

Supplement: Supplementary file 1 [file nutrients-13-02066-s001.zip › nutrients-1233298-supplementary.pdf]
